# Sagittal Abdominal Diameter, Waist Circumference, and BMI as Predictors of Multiple Measures of Glucose Metabolism: An NHANES Investigation of US Adults

**DOI:** 10.1155/2018/3604108

**Published:** 2018-06-19

**Authors:** Shelby A. Firouzi, Larry A. Tucker, James D. LeCheminant, Bruce W. Bailey

**Affiliations:** Department of Exercise Sciences, College of Life Sciences, Brigham Young University, Provo, UT 84602, USA

## Abstract

The objective was to compare associations between sagittal abdominal diameter (SAD), waist circumference, and BMI to the oral glucose tolerance test (OGTT), along with fasting glucose, HbA1c, and HOMA-IR, in a nationally representative sample of 3582 US adults. The study also analyzed the effect of multiple covariates on the anthropometric and glucose metabolism associations. A cross-sectional design was used. SAD was assessed using an abdominal caliper. All other data were collected following strict NHANES protocols. The OGTT was the primary variable used to index glucose metabolism. Fasting glucose, HbA1c, and HOMA-IR were also evaluated. Results showed that mean ± SE values were as follows: SAD: 22.3 ± 0.1 cm, waist circumference: 98.0 ± 0.4 cm, BMI: 28.6 ± 0.2 kg/m^2^, OGTT: 113.9 ± 1.0 mg/dL, fasting glucose: 99.6 ± 0.3 mg/dL, HbA1c: 5.4 ± 0.01%, and HOMA-IR: 3.2 ± 0.1. Compared to waist circumference and BMI, SAD consistently emerged as the best predictor of glucose metabolism, before and after adjusting for the covariates, and with the sample stratified by gender, race, or age. SAD was not a better predictor of OGTT among normal-weight adults or non-Hispanic Black adults. Due to the ease of taking SAD measurements, we recommend that healthcare providers use this simple method to more precisely predict diabetes risk, especially among overweight and obese adults.

## 1. Introduction

Recent findings show that over 29 million people in the United States have diabetes, with type 2 diabetes accounting for more than 90% of the cases [1, 2]. Over the past few decades, the prevalence of diabetes has increased by approximately 35% [3]. Among those with diabetes, approximately 1 in 4 remains undiagnosed, which is a serious obstacle to effective disease management [1, 2, 4]. Diabetes is not only a major risk factor for heart disease—the leading cause of death in the United States—but also the seventh-leading cause of death [5].

Obesity, a primary risk factor for type 2 diabetes, affects a significant portion of the United States population [5]. According to recent findings, almost 70% of US adults are overweight or obese [3]. If current trends continue, projections estimate that approximately 85% of the nation's adults could be overweight or obese by 2030 [6].

Central or abdominal obesity is associated with a myriad of metabolic disturbances, including glucose intolerance and insulin resistance, and has a greater association with type 2 diabetes than overall obesity [7]. Moreover, among the types of fat that account for abdominal obesity, visceral fat seems to have a more significant impact on diabetes and related conditions than subcutaneous fat [7].

Identifying valid, simple, and inexpensive screening tools for detecting diabetes risk has become a significant public health challenge, due to the increasing prevalence of diabetes and the large number of cases that go undiagnosed. Because a strong relationship exists between obesity, body fat distribution, and type 2 diabetes, anthropometric measurements can be used to screen and predict diabetes risk. BMI and waist circumference are among the most common measurement methods employed to help predict the relationship between body size, fat distribution, and glucose metabolism. However, these measurements are indirect approaches [8]. Computed tomography, magnetic resonance, and dual energy X-ray absorptiometry (DXA) are tools which can robustly assess visceral and abdominal fat. However, these methods tend to be expensive, and some produce radiation exposure [9].

The sagittal abdominal diameter (SAD) measurement, also referred to as “abdominal height,” has been introduced as a noninvasive method to index visceral fat [10]. SAD is measured with a subject laying in a supine position so that loose subcutaneous fat falls to the sides, and the harder, more rigid visceral fat stays in place to be measured via a caliper [9, 11]. The SAD measurement is valid and reliable no matter the body size of the individual, unlike the more traditional waist circumference measurement, which is more difficult to accurately and repeatedly measure in populations with overweight and obesity [12].

There are currently many methods to assess abdominal obesity, and there are also multiple methods to index glucose metabolism and diagnose diabetes, including the oral glucose tolerance test (OGTT), fasting blood glucose, hemoglobin A1c (HbA1c), and homeostasis model assessment (HOMA-IR) [13]. Overall, the OGTT is the gold standard, and research has consistently demonstrated the OGTT's unique ability to diagnose glucose-intolerant and diabetic cases that are missed when using other strategies [4, 14]. In general, HOMA-IR, an index of insulin resistance, does not diagnose diabetes status but is another widely recognized indicator of metabolic abnormalities used in large, epidemiologic studies [15, 16].

For the sagittal abdominal diameter (SAD) measurement to potentially operate as an index of abdominal obesity and help identify abnormal glucose metabolism, it should be evaluated in a national sample against the gold standard diagnostic tool for diabetes, the OGTT. Current research comparing the SAD against other anthropometric measurements (BMI, waist circumference, etc.), focusing on their capacity to quantify glucose metabolism using the OGTT, is lacking. To date, most investigations designed to study the sagittal abdominal diameter as it relates to OGTT results have employed special populations and small samples [12, 17–21]. The SAD has never been compared to other measures of abdominal obesity while using the OGTT in a large sample, representative of the US adult population. Such a study would allow findings to be generalized across various age groups, races, and body sizes within the United States.

The present study had multiple objectives. The first purpose was to compare the associations between SAD, waist circumference, and BMI to the gold standard assessment of glucose metabolism and the oral glucose tolerance test (OGTT), as well as other measures, including fasting glucose, HbA1c, and HOMA-IR, in a large, nationally representative sample of adults. Another purpose was to determine the effect of various covariates, including age, gender, and race, on the measures of obesity and abdominal obesity and glucose metabolism. Finally, the last aim was to compare the associations between the anthropometric variables and the measures of glucose metabolism across categories based on age, gender, race, and BMI separately.

## 2. Methods

### 2.1. Design

The present study employed a cross-sectional design. Data was obtained from the National Health and Nutrition Examination Survey (NHANES) (2011–2014). The associations between several anthropometric variables, including sagittal abdominal diameter (SAD), waist circumference, and BMI, and multiple measures of glucose metabolism, including the oral glucose tolerance test (OGTT), fasting glucose, HbA1c, and HOMA-IR, and the influence of covariates, were evaluated.

NHANES is a program of the National Center for Health Statistics, which is part of the Centers for Disease Control and Prevention. It is an extensive survey that annually assesses hundreds of variables related to health and nutrition. The target population for NHANES is the noninstitutionalized, civilian population of the United States [22]. Data from NHANES 2011-2012 and NHANES 2013-2014 were used for the present study. More details about the NHANES survey are available online [23].

### 2.2. Participants

NHANES uses a complex, multistage probability design to obtain a sample of the noninstitutionalized, civilian population in the United States. This sample is representative of the US adult population that is ≥20 years of age. NHANES-sampling procedures follow defined stages: (1) selection of primary sampling units (PSUs)—counties or small groups of counties; (2) selection of segments or blocks within PSUs; (3) selection of households within segments; and (4) selection of individuals within households [24, 25]. Between the years 2011 and 2014, 27,763 individuals were selected for NHANES from 60 different randomly selected study locations. Of these, 19,151 individuals were examined [24, 25]. Only a subsample of 4637 individuals who were examined during morning lab sessions was asked to complete an oral glucose tolerance test (OGTT). From the NHANES data, only subjects who had data on sagittal abdominal diameter (SAD), waist circumference, BMI (calculated from measures of height and weight), oral glucose tolerance test (OGTT), fasting glucose, HbA1c, and HOMA-IR were included in the present investigation. The number of subjects who met the inclusion criteria for this study was 3582 subjects.

The Institutional Review Board (IRB) for the National Center for Health Statistics (now referred to as the Ethics Review Board) approved the NHANES data collection and allowed the data files to be posted on their website for public use. NHANES received written informed consent from each survey participant prior to data collection [26].

### 2.3. Instrumentation and Measurement Methods

The following variables were studied in this investigation: age, gender, race, sagittal abdominal diameter (SAD), waist circumference, BMI (based on height and weight), oral glucose tolerance test (OGTT), fasting glucose, HbA1c, and HOMA-IR.

#### 2.3.1. Height

Standing height was measured using a fixed stadiometer with an adjustable headboard [27]. The final measurement was taken after telling the subject to stand as tall as possible, take a deep breath, and hold his/her position [27].

#### 2.3.2. Weight

Weight was determined using a Mettler Toledo digital weight scale. Each subject wore a standard examination gown consisting of a disposable shirt, pants, and slippers. If a subject weighed more than 440 pounds, the measurement was taken using two portable scales, and the subject stood with one foot on each scale. The two readings were then combined to approximate total body weight [27].

#### 2.3.3. Body Mass Index (BMI)

Body Mass Index (BMI) is a simple and universal index of weight, independent of height. The measure is based on an individual's weight and height. It is determined by taking the weight in kilograms divided by the height in meters squared (kg/m^2^). Underweight is classified as a BMI < 18.5, normal weight as a BMI between 18.5 and 24.99, overweight as a BMI between 25.0 and 29.99, and obese as a BMI ≥ 30. BMI was calculated using the measurements taken for weight and height [28].

#### 2.3.4. Sagittal Abdominal Diameter (SAD)

An abdominal caliper measured the distance between the front of the abdomen and the small of the back at the level of the iliac crest. Each subject was required to lie down on an examination table, bend his/her knees to a 90-degree angle, and keep the feet flat on the table. The arms were to remain crossed at the chest [27]. With the subject lying down, the examiner located the right iliac crest at the midaxillary line and marked the superior border of the right ilium with a line perpendicular to the table, using a cosmetic pencil. The examiner next located the same spot on the left side of the body and extended a measuring tape over the abdomen between the two marks, without compressing the skin, ensuring that the tape was still aligned perpendicular to the exam table [27]. A horizontal mark was drawn on the abdomen along the tape to allow for proper placement of the caliper. The caliper's lower arm was inserted beneath the small of the subject's back, so that the caliper arm and back touched. The upper arm of the caliper was lowered to approximately two centimeters above the subject's abdomen [27]. The subject was then instructed to slowly and gently inhale one breath, slowly exhale, and then pause. At this time, the upper caliper arm was slid down to lightly touch the abdomen without compressing it. At least two measurements were taken on each subject, and the average was used. Up to four measurements were taken if there was a difference greater than 0.5 cm between measurements, and the mean of the three closest values was calculated. Measurements were taken to the nearest 0.1 cm [27].

#### 2.3.5. Waist Circumference

Abdominal or waist circumference was measured directly against the skin of each subject, at the superior lateral border of the iliac crests. The subject crossed his/her arms and placed hands on opposite shoulders. The examiner stood on the right side of every participant, palpated the hip area to find the right ilium of the pelvis, and then drew a horizontal line directly above the most superior lateral border, using a cosmetic pencil [27]. A vertical line was also drawn at the midaxillary line to create a cross marking. A steel measuring tape was extended around the waist at the level of the measurement mark, with the examiner making sure that the tape stayed horizontal and parallel to the floor. The measurement tape was to be snug but not compress the skin. A single measurement was taken to the nearest 0.1 cm after the subject exhaled one normal breath [27].

#### 2.3.6. Oral Glucose Tolerance Test (OGTT)

The OGTT was performed on subjects who were examined during a morning lab session after a nine-hour fast. The exam was performed by a certified phlebotomist. The phlebotomist first administered an interview and a fasting questionnaire to screen for exclusion criteria and ensure fasting compliance. NHANES had seven exclusion criteria for the OGTT: pregnancy, hemophilia, and chemotherapy safety exclusions, fasting less than nine hours, using insulin or oral medication for diabetes, refusing phlebotomy, and not drinking the entire glucose solution within the prescribed time (10 minutes) [29–31]. After participants' initial fasting blood draw during their exam session, they were instructed to drink a 75-gram Trutol™ glucose solution within a maximum of 10 minutes. Participants continued to fast, and a second blood draw was taken two hours (±15 minutes) after drinking the Trutol solution [29–31].

#### 2.3.7. Plasma Fasting Glucose

The fasting glucose blood test was completed on all participants who were examined during a morning lab session following a nine-hour fast. The baseline test of the OGTT was used for the fasting glucose results [32, 33].

#### 2.3.8. Hemoglobin A1c (HbA1c)

The Tosoh Automated Glycohemoglobin Analyzer HLC-723G8 was used for in vitro quantitative measurement of hemoglobin A1c (HbA1c) [34].

#### 2.3.9. Homeostasis Model Assessment (HOMA-IR)

Insulin resistance was indexed using HOMA-IR (fasting insulin [*μ*U/mL] ∗ fasting glucose [mg/dL]/405). The methods for collecting fasting glucose data were described previously [32, 33]. Blood draws for fasting insulin were obtained by following the exact protocol as was used to obtain blood samples for fasting glucose. In the NHANES 2011-2012 data collection cycle, the Roche Elecsys 2010 immunoassay method was used to measure fasting insulin [35]. In the NHANES 2013-2014 cycle, a two-site immunoenzymatic assay was used to measure the insulin present in the blood sample, using the Tosoh AIA System Analyzer [36]. Because two different methods for collecting fasting insulin samples were employed in the 2011-2012 and 2013-2014 data cycles, the following regression equation was applied to the 2013-2014 insulin values to equalize them with the 2011-2012 values:

Insulin (Roche equivalent) = 10^∗∗^ (0.9765^∗^log10 (Tosoh insulin) + 0.07832) [37].

Unlike OGTT, fasting glucose, and HbA1c, HOMA-IR is not a direct measure of glucose metabolism. Rather, HOMA-IR is an index of insulin metabolism and insulin resistance. It was included with OGTT, fasting glucose, and HbA1c because HOMA-IR is widely used in epidemiologic research and it is moderately related to the measures of glucose metabolism and strongly associated with the anthropometric variables of the present study.

### 2.4. Statistical Analyses

NHANES findings are special because they can be generalized to the noninstitutionalized, civilian population of the United States. This broad level of generalization is possible because of NHANES' use of sophisticated sample weights. Each subject studied by NHANES receives a sample weight, a numerical value signifying the number of people in the US population represented by that individual. By using sample weights, NHANES produces unbiased national estimates, which account for unequal sample selection among different races, ages, genders, geographic locations, nonresponses, and independent population controls.

In the present investigation, means ± standard errors were provided for continuous variables to help describe the data. Results were adjusted for the NHANES complex sampling design, based on strata, primary sampling units, and individual sample weights. For the present study, sample weights were based on a subsample of fasting individuals who participated in the oral glucose tolerance test (OGTT) during the 2011-2012 and 2013-2014 data collection cycles.

Proc SurveyFreq was employed to estimate weighted frequencies, and Proc SurveyMeans was used to calculate weighted means, both representing the US population. Multiple linear regression using the Proc SurveyReg procedure was used to determine the relationship between each measure of abdominal obesity or obesity (i.e., sagittal abdominal diameter, waist circumference, and BMI) and each index of glucose metabolism (i.e., OGTT, fasting glucose, HbA1c, and HOMA-IR). As a result of the regression analyses, the amount of shared variance (*R*^2^) was reported for each association, along with corresponding *F* and *P* values. Steiger's *Z* was employed to determine the extent to which the correlated relationships differed significantly [38]. For example, Steiger's *Z* was used to test the extent to which the relationship between SAD and OGTT was stronger or weaker than the association between waist circumference and OGTT. To test the extent to which the relationships between the measures of abdominal obesity and glucose metabolism differed across various subgroups, including different age groups, races, men and women, and BMI categories, multiple regression analysis and Steiger's *Z* were used.

To test the degree to which the obesity and glucose metabolism relationships were mediated by age, gender, and race, these variables were controlled statistically using partial correlation and the Proc SurveyReg procedure. The least-squares means procedure was utilized to calculate adjusted means. Because of the correlations among the anthropometric measures that were employed as covariates in Model 3, the SAS VIF (variance inflation factor) option was used to test for multicollinearity. The most significant VIF tests showed “weak” levels of multicollinearity, according to Belsley et al. [39]. Hence, multicollinearity was not an issue.

If the glucose metabolism distributions (OGTT, fasting glucose, HbA1c, and HOMA-IR) deviated significantly from normal, the values were transformed by natural logarithm prior to modeling. To aid interpretation of the results, untransformed values were reported.

All *P* values were two-sided, and statistical significance was accepted when alpha was <0.05. The statistical analyses were computed using SAS Version 9.4 (SAS Institute Inc., Cary, NC).

## 3. Results

The final sample included 3582 participants, representing all noninstitutionalized, civilian US adults aged 20–84 years. The participants included men and women covering diverse racial categories and a wide range of BMIs. For the sample of women, the mean (±SE) age was 46.8 ± 0.6 years, average SAD was 21.7 ± 0.2 cm, and mean waist circumference and BMI were 96.0 ± 0.5 cm and 28.8 ± 0.2 kg/m^2^, respectively. For the glucose metabolism variables, among the women, mean (±SE) OGTT, fasting glucose, HbA1c, and HOMA-IR were 115.3 ± 1.4 mg/dL, 97.7 ± 0.4 mg/dL, 5.4 ± 0.01%, and 3.1 ± 0.1, respectively. Among the men, the mean (±SE) age was 45.1 ± 0.5 years, average SAD was 22.9 ± 0.1 cm, and mean waist circumference and BMI were 100.3 ± 0.4 cm and 28.3 ± 0.2 kg/m^2^, respectively. For the glucose metabolism variables in the sample of men, mean (±SE) OGTT, fasting glucose, HbA1c, and HOMA-IR were 112.5 ± 1.5 mg/dL, 101.7 ± 0.6 mg/dL, 5.4 ± 0.02%, and 3.4 ± 0.1, respectively.

Associations among the anthropometric variables were strong. Specifically, SAD and waist circumference were highly correlated (*r* = 0.945, *P* < 0.0001), whereas the relationships between waist circumference and BMI and between SAD and BMI were 0.906 (*P* < 0.0001) and 0.890 (*P* < 0.0001), respectively.

Correlations among the glucose metabolism variables in the full sample are displayed in Table 1. Among the measures of glucose metabolism, fasting glucose and HbA1c were correlated most strongly (*r* = 0.710, *P* < 0.0001) and the relationship between OGTT and fasting glucose was similar (*r* = 0.667, *P* < 0.0001).

As shown in Table 2, SAD was a significant predictor of OGTT, fasting glucose, HbA1c, and HOMA-IR, without any statistical controls and after adjusting for differences in age, gender, and race. SAD remained a significant predictor of each glucose metabolism variable after adjusting for the demographic variables, waist circumference, and BMI. Waist circumference and BMI were also significant predictors of each of the glucose metabolism variables with just age, gender, and race controlled. However, waist circumference and BMI were not significant predictors of the glucose measures when SAD and the other anthropometric variable were controlled statistically. SAD was a significantly better predictor of each glucose metabolism measure when compared to either waist circumference or BMI, before and after adjusting for the covariates (Table 2). Moreover, waist circumference was a statistically better predictor of the glucose measures than BMI in two-thirds of the comparisons (Table 2). Hence, overall, SAD was the best predictor of the glucose metabolism variables, followed by waist circumference, and then BMI.

Table 3 displays shared variances between the anthropometric and glucose metabolism variables with subjects stratified by gender. After adjusting for all covariates, SAD was again a significantly better predictor of each glucose metabolism variable compared to waist circumference or BMI in both men (*n* = 1784) and women (*n* = 1798). In the sample of men, SAD was always the best predictor of OGTT, fasting glucose, HbA1c, and HOMA-IR across all three of the statistical models. However, within the sample of women, SAD did not differ significantly from waist circumference as a predictor of fasting glucose and HbA1c when only age and race were controlled. In men, waist circumference remained a significantly better predictor than BMI for two-thirds of the comparisons. For women, no prominent pattern emerged in the comparisons between waist circumference and BMI. However, SAD remained the best predictor of glucose metabolism.

Table 4 features comparisons of shared variance by categories of BMI. For individuals in the normal-weight category (*n* = 1111), essentially no significant differences were established among the three anthropometric measurement methods. SAD was not a significantly better predictor of OGTT or any other glucose metabolism variable. In short, each anthropometric measure was virtually equal in its predictive utility of glucose metabolism when applied to a normal-weight US adult population.

As shown in Table 4, there were substantial differences in the relationships between the anthropometric measures and the glucose metabolism variables when applied to overweight (*n* = 1190) and obese (*n* = 1218) adults. In the overweight participants, SAD was again the best predictor of OGTT and the other glucose metabolism variables. However, waist circumference did not differ consistently from BMI as a predictor of glucose metabolism within the overweight group.

With the sample delimited to obese adults (Table 4), SAD was the best anthropometric predictor of OGTT—the gold standard diagnostic tool. For the other glucose metabolism measures (i.e., fasting glucose, HbA1c, and HOMA-IR), SAD remained the best predictor for eight of the nine comparisons. Unlike SAD, waist circumference and BMI did not persist as significant predictors of glucose metabolism when the demographic covariates and the other two anthropometric measures were controlled statistically.

Table 5 shows shared variances for subjects stratified by race. Within the non-Hispanic White population (*n* = 1526), SAD emerged again as the anthropometric measurement with the greatest predictive utility. For both Mexican Americans (*n* = 447) and Asians (*n* = 476), SAD was the best predictor across all glucose metabolism variables, after adjusting for all covariates. However, when the sample was delimited to non-Hispanic Blacks (*n* = 681), SAD lost some of its predictive edge when compared to waist circumference and BMI. In Blacks, BMI tended to be the worst predictor of the glucose metabolism measures. When analyzing the Other Hispanic group alone, after adjusting for all the covariates (Model 3), SAD was a better predictor of HbA1c and HOMA-IR compared to waist circumference and BMI.

Some differences appeared between SAD, waist circumference, and BMI as predictors of glucose metabolism when subjects were stratified by age, as shown in Table 6. Among adults 20–39 years old (*n* = 1350) and 60–84 years old (*n* = 967), SAD was the best predictor for OGTT and the other glucose metabolism variables, after controlling for all the covariates, but the relationship was not consistently significant with only age, gender, and race control. When the sample was delimited to middle-aged subjects (*n* = 1265), SAD was the best anthropometric predictor across all the statistical models.

## 4. Discussion

Despite significant advances in modern medicine, type 2 diabetes continues to plague millions of individuals in the US [1, 2]. In addition, about 70% of US adults are classified as overweight or obese, primary risk factors for type 2 diabetes [3, 5]. The strong association between obesity, abdominal obesity, and type 2 diabetes has been well-established in the scientific literature. This association has laid the foundation for further research to investigate the capability of various anthropometric measurements (SAD, waist circumference, and BMI) to distinguish individuals with abnormal glucose metabolism [7]. Some earlier investigations have shown that SAD, which can index abdominal fat specifically, may be a more effective predictor than waist circumference or BMI for predicting glucose intolerance and other metabolic disturbances [10, 40–42].

The purpose of this study was threefold. The first objective was to compare the associations between SAD, waist circumference, and BMI to the OGTT, the gold standard measurement of glucose metabolism, as well as to fasting glucose, HbA1c, and HOMA-IR, in a nationally representative sample of US adults. A second purpose was to analyze the effect of several covariates—age, gender, and race—on the relationships between the measures of obesity and abdominal obesity and the multiple indices of glucose metabolism used in the present investigation. The third purpose was to compare associations between the anthropometric and glucose metabolism variables across categories based on age, gender, race, and BMI separately.

While the differences were not large among the anthropometric measurements, overall, SAD consistently emerged as the best predictor of the OGTT results, as well as for the other glucose metabolism variables (fasting glucose, HbA1c, and HOMA-IR). Before and after adjusting for age, gender, race, and the other two anthropometric measures, SAD was a significantly better predictor of each glucose metabolism measure than both waist circumference and BMI. Moreover, waist circumference surfaced as a relatively consistent second-place measure in its predictive utility.

However, the results also presented certain conditions in which SAD was not the best predictor. Specifically, SAD was not a better predictor of OGTT than waist circumference or BMI within the subsample of normal-weight adults or among Black individuals.

Based on the results, the SAD measurement contains some unique quality allowing it to predict various indices of glucose metabolism better than the waist circumference and BMI measurements, despite the high correlations among the three variables. Consistently, SAD remained a significant predictor of glucose metabolism, even after adjusting for differences in waist circumference and BMI. The opposite was not true. This unique predictive quality seems to persist, even when subjects are stratified into different categories based on age, gender, race, and BMI.

The predictive power of the SAD measurement compared to waist circumference and BMI measurements was especially noteworthy when subjects were stratified by BMI category. While the anthropometric measurement method used appeared to have little significance in the normal-weight group, SAD's improved predictive edge was clearly apparent among overweight and obese individuals. These findings carry both statistical and practical significance, especially since more than two-thirds of adult Americans are overweight or obese [3, 4]. Because of the strong association between obesity and type 2 diabetes, it is highly likely that many individuals who are diagnosed with type 2 diabetes will also have either an overweight or obese BMI classification. Clinicians helping individuals with diabetic conditions will most likely be working with higher-weight populations rather than normal-weight individuals. Since the SAD measurement had the best predictive power among the overweight and obese, it would be most effective to apply this measurement tool to these groups.

Previous studies comparing the use of various anthropometric measurements in overweight and obese populations to predict glucose metabolism have supported the use of SAD [12, 41]. Gletsu-Miller et al. noted the potential effectiveness of using SAD measurements in these particular groups, as waist circumference can be a challenge to reliably measure due to larger girths, sagging skinfolds, and difficulty locating the correct landmarks for the measurement [12]. Furthermore, the waist circumference measurement includes both the depth (or height) and the width of the abdomen. On the other hand, the SAD measure focuses entirely on abdominal height—a better reflection of visceral adiposity—which is one of the best diagnostic criteria for metabolic abnormalities [41]. This difference probably explains, in part, why SAD is a better predictor of glucose metabolism than waist circumference or BMI in adults.

This study is the first investigation in the literature to compare SAD, waist circumference, and BMI as they relate to the OGTT, using a national sample. The findings of this investigation are consistent with the findings of previous studies in the literature, showing that SAD is a good predictor of indices of glucose metabolism [9, 41, 43–45]. In a study by Risérus et al. using 59 obese men, SAD had stronger correlations with all metabolic variables than both waist circumference and BMI, as well as the waist-to-hip ratio [41].

Only two previous studies have been conducted on a large national sample of US adults to investigate the relationship between SAD and glucose metabolic disturbances [42, 46]. While neither of these studies used the gold standard to measure glucose metabolism, OGTT, they corroborated the present results using HbA1c values [42, 46].

In one of these studies, Kahn et al. considered the role of physiological differences in adipose tissue on the effectiveness of the SAD measurement [42]. Adipose tissue can be classified as either superficial or deep, each with distinct physiological properties [42]. Advances in adipose tissue imaging suggest that the deep abdominal adipose tissue, which is associated with several cardiometabolic risk variables, is generally located near the anatomic midline, whereas superficial adipose tissue is more prominent on the sides of the abdomen [42]. As mentioned previously, because of the methods used to take a SAD measurement, SAD primarily incorporates deep—and therefore riskier—adipose tissue, more so than lateral superficial tissue [42].

In addition to SAD being able to measure deep adipose tissue more specifically, it can do so quickly and simply. Taking a SAD measurement requires only an examination table and portable caliper, and a patient can simply expose the midabdominal area, without having to change clothes or remove outer layers, shoes, jewelry, or medical appliances [46]. These accommodations allow individuals significant comfort and modesty, while still providing useful information to a healthcare provider.

A major strength of the present study was its large sample size representative of the US noninstitutionalized, civilian adult population. Because of the sampling method employed, the results are far more generalizable than previous studies investigating SAD and glucose metabolism. Another strength was the use of the OGTT, the gold standard measurement technique, as one of the glucose metabolism variables. Lastly, the present study analyzed and compared many different associations between anthropometric and glucose metabolism variables, which provided multiple outcomes across specific conditions from which to draw conclusions.

The present study also had some weaknesses. Because of its cross-sectional design, causality in the relationships between the anthropometric measurements and glucose metabolism could not be established. Additionally, there were many different relationships analyzed due to the large number of variables and statistical models included in the investigation. Because of the many comparisons, there was increased risk of type I error.

In summary, large differences were not found between SAD, waist circumference, and BMI. Notwithstanding the high correlations among these variables, SAD was the best anthropometric predictor of glucose metabolism compared to waist circumference and BMI when using OGTT, fasting glucose, HbA1c, and HOMA-IR. SAD was the best predictor across virtually all gender, BMI, racial, and age stratifications. SAD particularly had significant predictive utility among the overweight and obese, which are the primary populations struggling with type 2 diabetes.

## 5. Conclusion

In conclusion, the ability of SAD to index abdominal fat and high-risk obesity more precisely than waist circumference gives it a predictive edge over the more established anthropometric measurements to assist in detecting glucose abnormalities. The SAD measurement is not only meaningful but also quick to administer and noninvasive. Based on the findings of the present investigation, we recommend that healthcare providers consider using this simple, valid, and inexpensive measurement in clinical settings, especially among overweight and obese patients, to more precisely predict potential risk for prediabetes and diabetes, based on an OGTT or other indexes of glucose metabolism.

## Figures and Tables

**Table 1:** Correlations among the glucose metabolism variables.

Metabolic measure	OGTT	Fasting glucose	HbA1c	HOMA-IR
OGTT	1.000	0.667	0.596	0.409
Fasting glucose	0.667	1.000	0.710	0.515
HbA1c	0.596	0.710	1.000	0.384
HOMA-IR	0.409	0.515	0.384	1.000

Each correlation coefficient was statistically significant (*P* < 0.0001).

**Table 2:** Shared variance between the anthropometric variables and the glucose metabolism variables.

Metabolic measure	SAD			Waist circumference			BMI		
All subjects (*n* = 3582)	*R* ^2^	*F*	*P*	*R* ^2^	*F*	*P*	*R* ^2^	*F*	*P*
OGTT									
Model 1	0.092^a^	162.8	0.0001	0.073^b^	128.7	0.0001	0.057^c^	119.4	0.0001
Model 2	0.073^a^	163.5	0.0001	0.059^b^	164.0	0.0001	0.058^b^	162.5	0.0001
Model 3	0.014^a^	32.1	0.0001	0.001^b^	4.2	0.0483	0.000^c^	0.0	0.8989
Fasting glucose									
Model 1	0.146^a^	184.2	0.0001	0.130^b^	146.0	0.0001	0.085^c^	118.3	0.0001
Model 2	0.106^a^	192.2	0.0001	0.094^b^	158.1	0.0001	0.088^b^	157.7	0.0001
Model 3	0.011^a^	31.2	0.0001	0.000^b^	0.0	0.9376	0.000^b^	0.3	0.5996
HbA1c									
Model 1	0.090^a^	166.7	0.0001	0.068^b^	114.9	0.0001	0.048^c^	100.7	0.0001
Model 2	0.056^a^	129.1	0.0001	0.046^b^	110.1	0.0001	0.044^b^	97.7	0.0001
Model 3	0.010^a^	25.0	0.0001	0.001^b^	1.1	0.2958	0.000^c^	0.3	0.6094
HOMA-IR									
Model 1	0.423^a^	1051.6	0.0001	0.394^b^	683.6	0.0001	0.371^c^	837.3	0.0001
Model 2	0.426^a^	1198.1	0.0001	0.393^b^	761.9	0.0001	0.366^c^	871.5	0.0001
Model 3	0.031^a^	85.4	0.0001	0.001^b^	3.9	0.0583	0.000^c^	0.0	0.9733

*R* ^2^ includes only the shared variance between the metabolic measure and the anthropometric variable. Variance from the covariates is not included. Model 1: includes no covariates. Model 2: adjusted for age, gender, and race. Model 3: adjusted for age, gender, race, and the remaining two anthropometric variables. For example, Model 3 for OGTT and SAD adjusts for age, gender, race, waist circumference, and BMI. ^a,b,c^*R*^2^ values on the same row with the same superscript letter do not differ significantly (*P* > 0.05).

**Table 3:** Shared variance between the anthropometric variables and the glucose metabolism variables by gender.

Metabolic measure	SAD			Waist circumference			BMI		
	*R* ^2^	*F*	*P*	*R* ^2^	*F*	*P*	*R* ^2^	*F*	*P*
Men only (*n* = 1784)									
OGTT									
Model 1	0.125^a^	183.7	0.0001	0.102^b^	117.8	0.0001	0.076^c^	95.0	0.0001
Model 2	0.094^a^	149.6	0.0001	0.077^b^	125.0	0.0001	0.077^b^	136.4	0.0001
Model 3	0.018^a^	20.3	0.0001	0.003^b^	4.3	0.0500	0.001^c^	2.7	0.1115
Fasting glucose									
Model 1	0.141^a^	69.2	0.0001	0.127^b^	54.9	0.0001	0.098^c^	35.0	0.0001
Model 2	0.111^a^	67.6	0.0001	0.095^b^	51.5	0.0001	0.093^b^	42.8	0.0001
Model 3	0.014^a^	24.6	0.0001	0.001^b^	2.4	0.1297	0.001^b^	1.1	0.3119
HbA1c									
Model 1	0.088^a^	55.6	0.0001	0.068^b^	36.9	0.0001	0.048^c^	21.8	0.0001
Model 2	0.059^a^	44.1	0.0001	0.046^b^	30.2	0.0001	0.047^b^	25.8	0.0001
Model 3	0.014^a^	22.0	0.0001	0.003^b^	7.1	0.0121	0.001^c^	0.8	0.3690
HOMA-IR									
Model 1	0.454^a^	879.9	0.0001	0.421^b^	549.5	0.0001	0.398^c^	357.4	0.0001
Model 2	0.459^a^	765.6	0.0001	0.428^b^	489.3	0.0001	0.393^c^	363.4	0.0001
Model 3	0.031^a^	62.9	0.0001	0.001^b^	1.6	0.2191	0.000^c^	0.3	0.6114

Women only (*n* = 1798)									
OGTT									
Model 1	0.079^a^	64.9	0.0001	0.061^b^	59.0	0.0001	0.046^c^	44.4	0.0001
Model 2	0.060^a^	54.8	0.0001	0.050^b^	57.9	0.0001	0.050^b^	57.7	0.0001
Model 3	0.009^a^	8.7	0.0058	0.000^b^	0.6	0.4623	0.000^b^	0.0	0.9936
Fasting glucose									
Model 1	0.132^a^	168.9	0.0001	0.115^b^	143.8	0.0001	0.089^c^	140.1	0.0001
Model 2	0.105^a^	136.6	0.0001	0.096^a,b^	132.1	0.0001	0.088^a,b^	142.8	0.0001
Model 3	0.009^a^	10.3	0.0031	0.001^b^	1.1	0.3106	0.001^b^	0.9	0.3534
HbA1c									
Model 1	0.095^a^	163.8	0.0001	0.072^b^	148.0	0.0001	0.050^c^	112.3	0.0001
Model 2	0.054^a^	93.2	0.0001	0.048^a,b^	110.2	0.0001	0.043^a,b^	99.3	0.0001
Model 3	0.007^a^	7.2	0.0114	0.000^b^	0.1	0.7223	0.001^c^	2.0	0.1676
HOMA-IR									
Model 1	0.402^a^	489.2	0.0001	0.376^b^	342.4	0.0001	0.372^b^	399.0	0.0001
Model 2	0.389^a^	450.3	0.0001	0.359^b^	317.3	0.0001	0.350^b^	363.0	0.0001
Model 3	0.024^a^	53.0	0.0001	0.001^b^	1.3	0.2676	0.000^c^	0.7	0.3981

*R* ^2^ includes only the shared variance between the metabolic measure and the anthropometric variable. Variance from the covariates is not included. Model 1: includes no covariates. Model 2: adjusted for age and race. Model 3: adjusted for age, race, and the remaining two anthropometric variables. For example, Model 3 for OGTT and SAD adjusts for age, race, waist circumference, and BMI. ^a,b,c^*R*^2^ values on the same row with the same superscript letter do not differ significantly (*P* > 0.05).

**Table 4:** Shared variance between the anthropometric variables and the glucose metabolism variables by BMI category.

Metabolic measure	SAD			Waist circumference			BMI		
	*R* ^2^	*F*	*P*	*R* ^2^	*F*	*P*	*R* ^2^	*F*	*P*
Normal weight (*n* = 1111)									
OGTT									
Model 1	0.026^a^	19.0	0.0001	0.034^a^	25.7	0.0001	0.021^a^	11.5	0.0018
Model 2	0.005^a^	4.2	0.0494	0.008^a^	8.2	0.0074	0.011^a^	8.3	0.0070
Model 3	0.000^a^	0.0	0.8902	0.000^a^	0.3	0.5726	0.004^b^	3.21	0.0825
Fasting glucose									
Model 1	0.097^a^	180.1	0.0001	0.111^a^	113.5	0.0001	0.043^b^	33.5	0.0001
Model 2	0.025^a^	40.5	0.0001	0.030^a^	25.1	0.0001	0.018^a^	11.9	0.0016
Model 3	0.002^a,b^	1.0	0.3156	0.004^a^	2.22	0.1462	0.000^b^	0.2	0.6405
HbA1c									
Model 1	0.057^a^	47.4	0.0001	0.060^a^	50.6	0.0001	0.010^b^	6.7	0.0146
Model 2	0.002^a^	2.3	0.1413	0.002^a^	1.3	0.2724	0.000^a^	0.0	0.8754
Model 3	0.001^a^	1.2	0.2880	0.001^a^	0.6	0.4605	0.001^a^	1.0	0.3280
HOMA-IR									
Model 1	0.088^a^	70.6	0.0001	0.075^a^	51.8	0.0001	0.069^a^	104.6	0.0001
Model 2	0.122^a^	110.0	0.0001	0.109^a^	93.6	0.0001	0.073^b^	111.0	0.0001
Model 3	0.023^a^	10.4	0.0029	0.005^b^	2.9	0.0991	0.003^b^	3.2	0.0854

Overweight (*n* = 1190)									
OGTT									
Model 1	0.065^a^	62.2	0.0001	0.026^b^	16.2	0.0003	0.011^b^	6.4	0.0163
Model 2	0.033^a^	24.3	0.0001	0.011^b^	10.4	0.0029	0.014^b^	9.2	0.0048
Model 3	0.021^a^	11.9	0.0016	0.003^b^	2.5	0.1273	0.002^b^	1.5	0.2231
Fasting glucose									
Model 1	0.087^a^	97.1	0.0001	0.058^b^	53.3	0.0001	0.012^c^	9.5	0.0042
Model 2	0.032^a^	36.0	0.0001	0.014^b^	16.1	0.0003	0.013^b^	12.9	0.0011
Model 3	0.017^a^	16.8	0.0003	0.001^b^	1.1	0.3124	0.001^b^	1.1	0.3002
HbA1c									
Model 1	0.043^a^	42.8	0.0001	0.013^b^	16.5	0.0003	0.000^c^	0.5	0.4760
Model 2	0.011^a^	9.9	0.0036	0.002^b^	2.2	0.1456	0.001^b^	2.0	0.1656
Model 3	0.013^a^	13.1	0.0010	0.003^b^	4.3	0.0457	0.000^b^	0.1	0.7192
HOMA-IR									
Model 1	0.113^a^	82.4	0.0001	0.063^b^	63.7	0.0001	0.039^b^	32.1	0.0001
Model 2	0.123^a^	98.4	0.0001	0.070^b^	92.9	0.0001	0.041^c^	35.8	0.0001
Model 3	0.051^a^	22.8	0.0001	0.000^b^	0.0	0.9120	0.000^b^	0.2	0.6276

Obese (*n* = 1218)									
OGTT									
Model 1	0.040^a^	19.8	0.0001	0.016^b^	8.3	0.0070	0.007^b^	3.9	0.0574
Model 2	0.036^a^	20.3	0.0001	0.019^b^	13.0	0.0010	0.016^b^	9.9	0.0035
Model 3	0.023^a^	26.0	0.0001	0.002^b^	2.8	0.1024	0.001^b^	1.8	0.1906
Fasting glucose									
Model 1	0.088^a^	38.3	0.0001	0.061^b^	21.2	0.0001	0.023^c^	11.4	0.0019
Model 2	0.069^a^	35.6	0.0001	0.053^a^	24.1	0.0001	0.047^b^	23.3	0.0001
Model 3	0.015^a^	12.6	0.0012	0.000^b^	0.0	0.9791	0.000^b^	0.1	0.7686
HbA1c									
Model 1	0.072^a^	41.6	0.0001	0.037^b^	14.8	0.0005	0.021^b^	14.5	0.0006
Model 2	0.061^a^	42.9	0.0001	0.042^b^	24.4	0.0001	0.036^b^	23.3	0.0001
Model 3	0.020^a^	13.8	0.0008	0.000^b^	0.3	0.6214	0.001^b^	1.4	0.2494
HOMA-IR									
Model 1	0.241^a^	163.9	0.0001	0.199^b^	134.0	0.0001	0.145^c^	110.2	0.0001
Model 2	0.252^a^	218.2	0.0001	0.204^b^	146.6	0.0001	0.174^b^	153.5	0.0001
Model 3	0.045^a^	33.8	0.0001	0.001^b^	0.4	0.5182	0.000^b^	0.4	0.5290

*R* ^2^ includes only the shared variance between the metabolic measure and the anthropometric variable. Variance from the covariates is not included. Model 1: includes no covariates. Model 2: adjusted for age, gender, and race. Model 3: adjusted for age, gender, race, and the remaining two anthropometric variables. For example, Model 3 for OGTT and SAD adjusts for age, gender, race, waist circumference, and BMI. ^a,b,c^*R*^2^ values on the same row with the same superscript letter do not differ significantly (*P* > 0.05).

**Table 5:** Shared variance between the anthropometric variables and the glucose metabolism variables by race categories.

Metabolic measure	SAD			Waist circumference			BMI		
	*R* ^2^	*F*	*P*	*R* ^2^	*F*	*P*	*R* ^2^	*F*	*P*
Non-Hispanic White (*n* = 1526)									
OGTT									
Model 1	0.102^a^	92.8	0.0001	0.078^b^	75.6	0.0001	0.064^c^	75.9	0.0001
Model 2	0.076^a^	92.2	0.0001	0.059^b^	93.8	0.0001	0.061^b^	103.2	0.0001
Model 3	0.017^a^	18.4	0.0002	0.002^b^	5.3	0.0274	0.000^c^	0.3	0.5862
Fasting glucose									
Model 1	0.183^a^	116.2	0.0001	0.163^b^	87.5	0.0001	0.110^c^	81.1	0.0001
Model 2	0.125^a^	110.3	0.0001	0.112^b^	90.5	0.0001	0.107^b^	98.9	0.0001
Model 3	0.011^a^	13.6	0.0008	0.000^b^	0.0	0.9554	0.000^b^	0.3	0.5939
HbA1c									
Model 1	0.104^a^	86.6	0.0001	0.083^b^	58.0	0.0001	0.050^c^	38.9	0.0001
Model 2	0.060^a^	70.0	0.0001	0.049^b^	56.8	0.0001	0.047^b^	49.8	0.0001
Model 3	0.011^a^	12.6	0.0012	0.001^b^	0.7	0.4217	0.000^c^	0.1	0.7891
HOMA-IR									
Model 1	0.445^a^	602.9	0.0001	0.409^b^	416.8	0.0001	0.381^c^	465.7	0.0001
Model 2	0.443^a^	626.4	0.0001	0.404^b^	432.4	0.0001	0.380^c^	495.2	0.0001
Model 3	0.035^a^	66.8	0.0001	0.000^b^	0.6	0.4314	0.000^b^	0.2	0.6699

Non-Hispanic Black (*n* = 681)									
OGTT									
Model 1	0.078^a^	87.2	0.0001	0.066^a^	73.3	0.0001	0.037^b^	32.0	0.0001
Model 2	0.056^a^	60.0	0.0001	0.056^a^	61.0	0.0001	0.047^a^	40.8	0.0001
Model 3	0.002^a^	2.3	0.1432	0.002^a^	2.5	0.1273	0.001^a^	0.9	0.3491
Fasting glucose									
Model 1	0.105^a^	121.3	0.0001	0.096^a^	77.4	0.0001	0.067^b^	61.1	0.0001
Model 2	0.087^a^	126.9	0.0001	0.090^a^	96.8	0.0001	0.084^a^	110.2	0.0001
Model 3	0.001^a,b^	0.5	0.4853	0.002^a^	1.3	0.2701	0.000^b^	0.4	0.5174
HbA1c									
Model 1	0.082^a^	78.5	0.0001	0.065^b^	55.0	0.0001	0.049^c^	35.0	0.0001
Model 2	0.060^a^	92.2	0.0001	0.053^a^	71.8	0.0001	0.052^a^	59.9	0.0001
Model 3	0.007^a^	9.6	0.0043	0.000^b^	0.4	0.5400	0.000^b^	0.1	0.7670
HOMA-IR									
Model 1	0.338^a^	204.9	0.0001	0.346^a^	212.6	0.0001	0.322^a^	214.0	0.0001
Model 2	0.330^a^	255.7	0.0001	0.329^a^	243.6	0.0001	0.295^b^	214.5	0.0001
Model 3	0.009^a^	10.3	0.0032	0.007^a^	9.6	0.0044	0.000^b^	0.2	0.6462

Mexican American (*n* = 447)									
OGTT									
Model 1	0.107^a^	18.1	0.0003	0.083^b^	14.9	0.0007	0.073^b^	17.1	0.0004
Model 2	0.088^a^	18.7	0.0002	0.069^b^	15.8	0.0006	0.059^b^	17.9	0.0003
Model 3	0.025^a^	7.3	0.0126	0.000^b^	0.1	0.7803	0.003^c^	3.6	0.0701
Fasting glucose									
Model 1	0.105^a^	24.4	0.0001	0.072^b^	18.6	0.0002	0.049^c^	14.2	0.0010
Model 2	0.075^a^	18.9	0.0002	0.052^b^	14.7	0.0008	0.049^b^	17.9	0.0003
Model 3	0.030^a^	13.0	0.0014	0.003^b^	2.4	0.1348	0.002^b^	1.2	0.2928
HbA1c									
Model 1	0.083^a^	14.4	0.0009	0.066^a,b^	13.3	0.0013	0.047^b^	11.8	0.0022
Model 2	0.057^a^	11.9	0.0021	0.047^a^	11.4	0.0025	0.042^a^	12.5	0.0017
Model 3	0.011^a^	4.6	0.0419	0.000^b^	0.0	0.8560	0.001^b^	0.6	0.4656
HOMA-IR									
Model 1	0.408^a^	478.8	0.0001	0.384^a,b^	397.4	0.0001	0.365^b^	391.2	0.0001
Model 2	0.414^a^	433.4	0.0001	0.385^a,b^	388.8	0.0001	0.368^b^	481.7	0.0001
Model 3	0.023^a^	7.7	0.0107	0.001^b^	0.6	0.4500	0.001^b^	0.3	0.5647

Other Hispanic (*n* = 358)									
OGTT									
Model 1	0.132^a^	21.6	0.0001	0.126^a^	24.8	0.0001	0.089^b^	15.9	0.0004
Model 2	0.084^a^	23.0	0.0001	0.082^a^	24.3	0.0001	0.070^a^	19.3	0.0001
Model 3	0.005^a^	2.8	0.1054	0.002^a,b^	2.8	0.1063	0.000^b^	0.2	0.6289
Fasting glucose									
Model 1	0.152^a^	34.6	0.0001	0.126^b^	31.3	0.0001	0.055^b^	12.6	0.0014
Model 2	0.090^a^	33.5	0.0001	0.070^a,b^	27.7	0.0001	0.046^b^	18.1	0.0002
Model 3	0.032^a,c^	13.4	0.0010	0.001^b^	0.8	0.3813	0.018^c^	15.6	0.0005
HbA1c									
Model 1	0.076^a^	14.9	0.0006	0.050^b^	9.7	0.0043	0.026^c^	5.7	0.0244
Model 2	0.036^a^	8.2	0.0078	0.019^b^	4.7	0.0398	0.017^b^	4.9	0.0352
Model 3	0.030^a^	12.5	0.0015	0.005^b^	2.9	0.1019	0.002^b^	1.7	0.2033
HOMA-IR									
Model 1	0.494^a^	211.3	0.0001	0.479^a^	218.3	0.0001	0.389^b^	86.9	0.0001
Model 2	0.461^a^	200.5	0.0001	0.446^a^	221.5	0.0001	0.381^b^	96.5	0.0001
Model 3	0.028^a^	19.8	0.0001	0.012^b^	8.1	0.0083	0.002^c^	1.3	0.2692

Asian (*n* = 476)									
OGTT									
Model 1	0.095^a^	39.0	0.0001	0.099^a^	37.2	0.0001	0.086^a^	37.4	0.0001
Model 2	0.075^a^	48.0	0.0001	0.072^a^	40.8	0.0001	0.070^a^	41.3	0.0001
Model 3	0.004^a^	2.1	0.1624	0.000^b^	0.2	0.6762	0.001^b^	0.9	0.3540
Fasting glucose									
Model 1	0.158^a^	85.0	0.0001	0.150^a^	78.3	0.0001	0.110^b^	50.8	0.0001
Model 2	0.085^a^	29.2	0.0001	0.078^a^	27.5	0.0001	0.069^a^	26.5	0.0001
Model 3	0.009^a^	4.4	0.0439	0.001^b^	0.4	0.5508	0.000^b^	0.1	0.8067
HbA1c									
Model 1	0.104^a,b^	57.2	0.0001	0.121^a^	50.2	0.0001	0.092^b^	39.9	0.0001
Model 2	0.069^a,b^	27.6	0.0001	0.078^a^	24.7	0.0001	0.067^b^	29.8	0.0001
Model 3	0.000^a^	0.1	0.7153	0.006^b^	1.7	0.2056	0.000^a^	0.0	0.9834
HOMA-IR									
Model 1	0.442^a^	221.0	0.0001	0.413^a,b^	218.5	0.0001	0.398^b^	191.8	0.0001
Model 2	0.441^a^	211.6	0.0001	0.410^a,b^	206.5	0.0001	0.377^b^	185.6	0.0001
Model 3	0.034^a^	55.6	0.0001	0.003^b^	3.0	0.0944	0.000^c^	0.3	0.6022

**Table 6:** Shared variance between the anthropometric variables and the glucose metabolism variables by age category.

Metabolic measure	SAD			Waist circumference			BMI		
	*R* ^2^	*F*	*P*	*R* ^2^	*F*	*P*	*R* ^2^	*F*	*P*
20–39 years (*n* = 1350)									
OGTT									
Model 1	0.089^a^	125.5	0.0001	0.080^a^	98.2	0.0001	0.081^a^	94.2	0.0001
Model 2	0.092^a^	126.7	0.0001	0.080^b^	92.7	0.0001	0.079^b^	89.9	0.0001
Model 3	0.011^a^	12.3	0.0014	0.000^b^	0.5	0.4835	0.000^b^	0.1	0.7204
Fasting glucose									
Model 1	0.145^a^	169.7	0.0001	0.135^a^	128.9	0.0001	0.103^b^	123.8	0.0001
Model 2	0.121^a^	137.6	0.0001	0.114^a^	105.3	0.0001	0.101^b^	108.1	0.0001
Model 3	0.010^a^	12.6	0.0012	0.001^b^	2.1	0.1532	0.001^b^	2.2	0.1474
HbA1c									
Model 1	0.071^a^	56.9	0.0001	0.062^b^	52.1	0.0001	0.055^b^	43.2	0.0001
Model 2	0.052^a^	38.7	0.0001	0.050^a^	38.6	0.0001	0.045^a^	33.5	0.0001
Model 3	0.003^a^	2.3	0.1367	0.001^b^	0.9	0.3592	0.000^c^	0.4	0.5333
HOMA-IR									
Model 1	0.429^a^	821.3	0.0001	0.404^b^	589.8	0.0001	0.384^c^	705.6	0.0001
Model 2	0.441^a^	1348.7	0.0001	0.417^b^	917.3	0.0001	0.383^c^	959.0	0.0001
Model 3	0.027^a^	58.4	0.0001	0.003^b^	9.1	0.0049	0.001^c^	2.0	0.1703

40–59 years (*n* = 1265)									
OGTT									
Model 1	0.067^a^	59.1	0.0001	0.049^b^	37.2	0.0001	0.049^b^	37.7	0.0001
Model 2	0.078^a^	59.3	0.0001	0.059^b^	43.0	0.0001	0.052^b^	41.5	0.0001
Model 3	0.024^a^	30.4	0.0001	0.002^b^	1.6	0.2148	0.001^b^	1.2	0.2775
Fasting glucose									
Model 1	0.115^a^	124.0	0.0001	0.100^b^	114.1	0.0001	0.070^c^	75.6	0.0001
Model 2	0.101^a^	128.1	0.0001	0.087^b^	117.3	0.0001	0.078^b^	93.7	0.0001
Model 3	0.014^a^	16.7	0.0003	0.000^b^	0.0	0.8585	0.000^b^	0.3	0.5693
HbA1c									
Model 1	0.070^a^	72.0	0.0001	0.055^b^	57.5	0.0001	0.050^b^	42.3	0.0001
Model 2	0.069^a^	68.7	0.0001	0.059^b^	64.7	0.0001	0.049^b^	45.8	0.0001
Model 3	0.011^a^	12.5	0.0013	0.000^b^	0.0	0.8751	0.001^c^	1.9	0.1837
HOMA-IR									
Model 1	0.411^a^	344.9	0.0001	0.381^b^	244.5	0.0001	0.349^c^	221.1	0.0001
Model 2	0.415^a^	341.9	0.0001	0.380^b^	247.6	0.0001	0.351^c^	226.7	0.0001
Model 3	0.031^a^	29.2	0.0001	0.000^b^	0.4	0.5168	0.000^b^	0.2	0.6405

60–84 years (*n* = 967)									
OGTT									
Model 1	0.069^a,c^	18.9	0.0001	0.050^b^	15.6	0.0004	0.063^c^	22.2	0.0001
Model 2	0.090^a^	21.3	0.0001	0.081^a^	25.4	0.0001	0.082^a^	26.2	0.0001
Model 3	0.005^a^	1.7	0.2053	0.000^b^	0.0	0.8875	0.002^c^	1.2	0.2732
Fasting glucose									
Model 1	0.127^a^	14.6	0.0006	0.117^a^	13.4	0.0009	0.111^a^	12.9	0.0011
Model 2	0.124^a^	15.2	0.0005	0.115^a^	13.9	0.0007	0.122^a^	14.0	0.0007
Model 3	0.004^a^	2.8	0.1035	0.000^b^	0.0	0.9111	0.004^a^	3.2	0.0834
HbA1c									
Model 1	0.062^a,c^	13.3	0.0009	0.033^b^	5.7	0.0229	0.054^c^	11.1	0.0022
Model 2	0.073^a,c^	17.5	0.0002	0.049^b^	9.3	0.0047	0.062^c^	12.2	0.0014
Model 3	0.021^a^	12.8	0.0011	0.010^b^	4.6	0.0407	0.002^c^	1.6	0.2227
HOMA-IR									
Model 1	0.445^a^	265.6	0.0001	0.407^b^	271.1	0.0001	0.396^b^	173.0	0.0001
Model 2	0.442^a^	261.6	0.0001	0.415^b^	274.9	0.0001	0.401^b^	185.8	0.0001
Model 3	0.021^a^	30.8	0.0001	0.002^b^	2.1	0.1545	0.003^b^	1.8	0.1913

*R* ^2^ includes only the shared variance between the metabolic measure and the anthropometric variable. Variance from the covariates is not included. Model 1: includes no covariates. Model 2: adjusted for gender and race. Model 3: adjusted for gender, race, and the remaining two anthropometric variables. For example, Model 3 for OGTT and SAD adjusts for gender, race, waist circumference, and BMI. ^a,b,c^*R*^2^ values on the same row with the same superscript letter do not differ significantly (*P* > 0.05).

## Data Availability

All data used in the present study are available online as part of the National Health and Nutrition Examination Survey (NHANES). The data are free and can be accessed by using the following Centers for Disease Control and Prevention website: https://wwwn.cdc.gov/nchs/nhanes/Default.aspx.
